# Human Papillomavirus Coinfection in the Cervical Intraepithelial Lesions and Cancer of Mexican Patients

**DOI:** 10.1155/2020/4542320

**Published:** 2020-11-13

**Authors:** Juan Ramón Padilla-Mendoza, Lucía Angélica Gómez-López, Mavil López-Casamichana, Elisa Irene Azuara-Liceaga, Enoc Mariano Cortés-Malagón, Lilia López-Cánovas, Octavio Daniel Reyes-Hernández, Mario Alberto Rodríguez, José Bonilla-Delgado, Israel López-Reyes

**Affiliations:** ^1^Departamento de Infectómica y Patogénesis Molecular, Centro de Investigación y de Estudios Avanzados (CINVESTAV-IPN), Ciudad de México, Mexico; ^2^Posgrado en Ciencias Genómicas, Universidad Autónoma de la Ciudad de México (UACM), Plantel Del Valle, Ciudad de México, Mexico; ^3^Unidad de Genética y Cáncer, Hospital Juárez de México (HJM), Ciudad de México, Mexico; ^4^Facultad de Estudios Superiores Zaragoza, Universidad Nacional Autónoma de México (UNAM), Ciudad de México, Mexico; ^5^Universidad Autónoma de la Ciudad de México (UACM), Plantel Cuautepec, Ciudad de México, Mexico

## Abstract

According to their oncogenic properties, Human Papillomaviruses (HPVs) are classified into two types: Low-Risk (LR-HPVs) and High-Risk Human Papillomaviruses (HR-HPVs). The immune system naturally controls the majority of HPV infections; however, when the HR-HPV infection is persistent, the risk of developing cervical cancer increases. Previous studies indicate that multiple-infection or coinfection with HR-HPV occurs frequently and can potentiate the development of cervical lesions. This study aimed to establish the HPV coinfection rate in squamous intraepithelial lesions from Mexican patients. For HPV detection, we performed PCR on 55 cervical lesions diagnosed by colposcopy. We detected the presence of HPV infection in 87.27% (48/55) of the lesions; interestingly, HPV coinfection was observed in 70.83% (34/48) of these samples. We also evaluated HPV infection in adjacent areas without morphological changes from 25 samples. The results showed that 80% (20/25) of these were HPV-positive and, curiously, all presented HPV-16 infection. In conclusion, our results revealed a high prevalence of HPV coinfection in cervical lesions in Mexican patients, and these results contribute to future research focused on the role that HPV coinfection plays in the development of cervical cancer.

## 1. Introduction

Cervical cancer (CC) is the second type of cancer among women in developing countries and is the primary neoplasm associated with Human Papillomavirus (HPV) infection [1]. Late detection of CC and its precursor lesions can lead to the death of these women, making it a public health problem [2]. Persistent infection in the cervix with High-Risk Human Papillomaviruses (HR-HPV) produces precancerous lesions that eventually progress to CC [3–5].

There are more than 200 HPV types, of which 12-15 are classified as HR-HPV [6, 7]. Cervical infection with more than one HPV genotype (coinfection) is common in 20-50% of HPV-infected women, especially among young women [8]. Infection with multiple HPV types is often considered a risk factor leading to the development of CC [9]. However, the relationship between multiple HPV-type infections and the progression of CC is questionable and remains unclear. The main debate lies in whether these infections occur due to affinity among certain HPV types or randomly. The establishment of persistent HPV infection is challenging; although, coinfection is probably involved, perhaps by means of competitive or cooperative interactions between HPV types, which could increase or decrease the risk of the progression of CC. Despite several contradictions, few studies have evaluated the relationship between multiple HPV-type infections and the risk of cervical disease [10].

In this study, we analyzed multiple HPV types of infection in biopsies from Mexican patients diagnosed with cervical lesions and CC. Our data revealed the presence of multiple HPV-type infections in the majority of samples with lesion (70.83%), but only 30% in adjacent tissue without morphological changes.

## 2. Materials and Methods

### 2.1. Samples

Biopsies were obtained from Mexican women seen at Hospital IMSS “La Raza” (Mexico City) and “Hospital Juárez de México” (Mexico City) and who were pathologically diagnosed with Atypical Squamous Cells of Undetermined Significance (ASCUS), Low-grade Squamous Intraepithelial Lesion (LSIL), High-grade Squamous Intraepithelial Lesion (HSIL), or Cervical Cancer (CC). The present study conducted according to the Declaration of Helsinki for Medical Protocol and Ethics, and the Institutional Committee of Research and Ethics approved the study (registration no. HMJ 2231/13-B). Expert colposcopists performed all examinations, and samples were classified according to the International Federation of Gynecology and Obstetrics (FIGO) [11]. Fifty-five biopsies of the cervical lesions from patients with an age range between 20 and 65 years (8 ASCUS, 25 LSIL, 11 HSIL, and 11 CC) plus 25 biopsies of adjacent regions without morphological changes, for a total of eighty samples, were analyzed.

Biopsies were placed in Tissue-Tek medium (Sakura Finetek, USA) for their preservation and stored at −80°C prior to DNA extraction.

### 2.2. HPV Detection and Genotyping

Samples were mechanically disrupted and subsequently homogenized in lysis buffer for DNA extraction according to the established protocol by the DNA/RNA All Prep Kit (Qiagen®, USA). Amplification of the *gapdh* gene was performed to test sample sufficiency employing the template 100 ng of DNA and targeting a 140-bp sequence with the following primers: forward: 5′-AGGTGACACTATAGAATAACCGTCAAGGC-3′; and reverse: 5′-GTACGHACTCACTATAGGGATGGTGGTGA-3′. To detect HPV, we performed PCR assays using the PGMY09/PGMY11 primers (MY11: 5′-GCMCAGGGWCATAAYAATGG-3′; MY09: 5′-CGTCCMARRGGAWACTGATC-3′–) targeting a 450-bp sequence of the *L1* gene [12]. According to the established protocol, genotyping was performed using the Kit CLART® HPV2 (Genomica®, Spain). This kit, through multiplex PCR and low density arrays, detects the following: 35 HPV types, including 14 high-risk (HR-HPV) (HPV-16, HPV-18, HPV-31, HPV-33, HPV-35, HPV-39, HPV-45, HPV-51, HPV-52, HPV-56, HPV-58, HPV-59, HPV-66, and HPV-68B), four probably High-Risk (HPV-26, HPV-53, HPV-73, and HPV-82), and 17 Low-Risk (LR-HPV) (HPV-6, HPV-11, HPV-40, HPV-42, HPV-43, HPV-44, HPV-54, HPV-61, HPV-62, HPV-70, HPV-71, HPV-72, HPV-81, HPV-83, HPV-84, HPV-85, and HPV-89).

### 2.3. Statistical Analysis

A woman (biopsy) infected with more than one HPV genotype was considered to have a multiple HPV-type infection or coinfection. To determine Student's *t*-test independence among variables, analysis of variance (ANOVA) was utilized to compare groups. Odds ratios (OR), calculated with the Medcalc® and StatSoft 8.0® software, were determined to estimate the association of genotypes with cervical carcinoma. *P* values of < 0.05 were considered statistically significant.

## 3. Results

This study included biopsies from 55 patients with a mean age of 42.5 years. Samples were grouped according to their pathological diagnosis. Thus, we analyzed eight biopsies diagnosed with ASCUS (15%), 25 biopsies diagnosed with LSIL (45%), 11 biopsies diagnosed with HSIL (20%), and 11 biopsies diagnosed with CC (20%). Moreover, from 25 of these biopsies (6 with LSILs, 10 with HSILs, and 9 with CCs), we obtained a sample of adjacent tissue without morphological changes.

**Figure 1:**
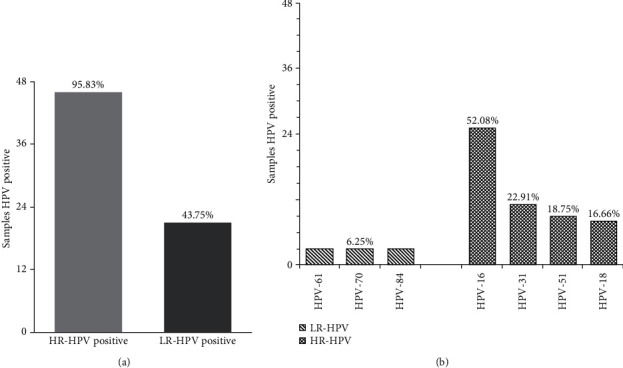
HPV groups present in samples with cervical lesion. The figure shows the number of positive samples by HPV group (a); moreover, subsection (b) depicts the number of positive samples by HPV genotype that was more present.

Overall, 48 of 55 biopsies with cervical lesions were HPV positive; therefore, HPV prevalence was 87.27%; 95.83% (46/48) of HPV-positive biopsies contained HR-HPV (Figure 1(a)) corresponding to 13 genotypes, among which the most frequent were HPV-16 (52.08%), HPV-31 (22.91%), HPV-51 (18.75%), and HPV-18 (16.66%) (Figure 1(b)). On the other hand, 43.75% (21/48) of the HPV-positive samples presented LR-HPV (Figure 1(a)) with 12 different genotypes, among which the most frequent were HPV-61, HPV-70, and HPV-84 (6.25% for each genotype) (Figure 1(b)).

Interestingly, we found that 70.83% (34/48) of HPV-positive samples presented more than one HPV genotype (coinfection), while the remained only had a single infection (Table 1). Of the samples with coinfection, 55.88% (19/34) demonstrated a mixed coinfection (infection with HR- and LR-HPV). The remaining 44.12% (15/34) displayed a simple coinfection (genotypes of the same HPV group), of which 13 (38.23%) were coinfection with HR-HPV genotypes, whereas only two (5.88%) exhibited coinfection with LR-HPV genotypes (Table 1). HPV-31 and HPV-51 were the most common genotypes in LSIL, while HPV-16 was the main genotype in CC (data not shown).

In that, the most frequent HR-HPVs in CC at worldwide are HPV-16 and HPV-18 [13]; we evaluated the combination of these genotypes with other HR-HPV genotypes. Of the 13 samples with HR-HPV simple coinfection, seven (53.84%) showed the presence of HPV-16, whereas coinfection of HPV-18 with other HR-HPV genotypes was detected in two samples (15.38%) (Table 1). On the other hand, of the 19 samples with mixed coinfection, five (26.31%) showed the presence of HPV-16, and five (26.31%) showed the presence of HPV-18 (Table 1).

The OR statistical analysis revealed that coinfection with more than one HPV type has a probability of 97.6% of developing LSIL (Table 2). This estimate is considerably higher than those entertaining the likelihood of producing severe dysplasia, such as HSIL and CC (18.6% and 5.8%, respectively) (Table 2). On the other hand, we selected the three most prevalent HPV genotypes in our study (HPV-16, HPV-31, and HPV-51) to estimate their probability of developing a cervical lesion. OR analysis showed that the infection with HPV-31 and HPV-51 genotypes increases 5.5- and 7-fold, respectively, the risk of developing LSIL, whereas infection with the HPV-16 genotype increases the risk of developing cervical cancer by 10.3 times (Table 3). In addition, the analysis of the 25 biopsies from adjacent regions in terms of cervical lesions without morphological changes demonstrated that 80% of these (20/25) were HPV-positive. Interestingly, this percentage is very similar to that of HPV-positive biopsies with cervical lesion (87.27%).

From these biopsies, all from patients with HSIL (10/10), 89% (8/9) from patients with CC, and 33% (2/6) from patients with LSIL were HPV-positive. Although the percentages of HPV-positive samples were similar between cervical lesions and adjacent regions without morphological changes, the proportion of HPV coinfection was different. Samples without morphological changes but that were HPV-positive exhibited only HR-HPV genotypes with eight different HPV. All of these samples contained HPV-16 (Table 4); 70% (14/20) demonstrated a single infection, and 30% (6/20) displayed coinfection (one sample with mixed coinfection and five with simple coinfection). Interestingly, all biopsies of the region adjacent to CC that were infected with HPV (*n* = 8) contained a single infection; while 40% (4/10) of biopsies without morphological changes from patients diagnosed with HSIL presented coinfection, and one of these showed four different HR-HPV genotypes. Finally, the two biopsies without morphological changes from patients diagnosed with LSIL and who were HPV-infected revealed coinfection (Table 4).

In summary, our data demonstrated a high prevalence of multiple HPV-types infection in Mexican patients with different grades of cervical lesion, a condition that could participate in the development of the diverse injuries.

## 4. Discussion

Cervical cancer is the fourth most frequent neoplasm worldwide, affecting 570,000 women and causing more than 311,000 deaths in the last year [14]. In Mexico, CC is the third most prevalent cancer in women, with 7,689 new cases reported in 2018 [15]. Although HPV is the causal agent for the development of cervical precancerous lesions and CC, the role of infections with different types of HPV or coinfections remains unclear.

Here, we analyzed the presence of HPV single infection and coinfection in Mexican patients with different cervical lesions. Our data showed an 87.2% HPV detection rate in these samples, while previous studies reported rates as high as 98% [16], and the global prevalence of HPV infection in women is 65% [17]. The disparity of these data with our results could perhaps be explained by the size of our sample (*n* = 55). Moreover, the percentage of negativity (12.73%) in our samples could be related to the PCR type employed (simple PCR with primers MY09/MY11). In addition, we cannot rule out that negative cases in our study may be applicable to viral load, which could be below the severity of the technique. Other studies have concluded that utilizing a nested PCR using the MY09/MY11 and GP5+/GP6+ primers can significantly increase the sensitivity from 10 to 100 times, compared with the simple PCR employing only the MY09/MY11 primers [12]. In this respect, a multicenter study in Spain and Colombia using the MY09/MY11 primers identified viral DNA in 75% of cases; the rest of the samples were reanalyzed using the nested PCR system and achieved a 20% increase in the range of detection [18].

Nevertheless, we found an HPV incidence greater than or equal to 80%, regardless of the injury type diagnosed. However, we did not detect a correlation between the presence of infection and lesion type (*P* = 0.235). The data suggest that, although HPV infection is involved in the development of cervical lesions, it is not sufficient to predict the injury type that could be produced. In agreement with previous reports, these results suggest that other factors might be implicated in the development of each lesion type, including the viral genotype and the presence of more than one viral type [19]. The genotypes distribution found in our study is similar to that previously reported in Mexico [20]. We found that HPV-16 and HPV-18 are the most common genotypes, which is consistent with previous studies carried out with Mexican patients [17, 21]. Other HPV types detected were HPV-31, HPV-51, HPV-18, HPV-58, and HPV-53, which are the most prevalent in different states of the Mexican Republic [22–26]. However, worldwide, there are geographic differences in the frequency of HPV types; for instance, HPV-52 and HPV-58 were the most prevalent types in HSIL and CC in China [27, 28]. Indeed, these genotypes are more common than HPV-43, HPV-31, and HPV-33 throughout Asia [27], whereas in Mali, Africa, HPV-51 and HPV-73 are more frequent than HPV-16 and HPV-18 [21].

Sasagawa et al. [28] found, according to their OR analysis, that HPV-11, HPV-39, HPV-42, HPV-44, HPV-53, HPV-59, HPV-62, and HPV-66 were associated with LSIL, HPV-33, HPV-35, and HPV-56 that were associated to HSIL, while HPV-16, HPV-18, HPV-31, HPV-51, HPV-52, and HPV-58 were associated to invasive carcinoma. In our study, the most common genotypes in LSIL were HPV-31 (40%) and HPV-51 (35%), which are risk factors for developing this injury (OR = 5.5 and OR = 7, respectively). In the same manner, HPV-16, the most frequent genotype in patients diagnosed with cervical cancer, increased by 10.3 times the likelihood of developing this lesion. However, we utilized a low-density microarray to detect the 35 viral genotypes most prevalent worldwide; therefore, viral types undetectable by this microarray could be present in our samples.

On the other hand, we observed a frequency of coinfection of 70.83%, which is consistent with previous reports. For instance, García-Espinosa et al. [29] reported a coinfection of 78.8% in Equatorial Guinea. Another study conducted in Japanese women associated multiple HPV infections with LSIL, HSIL, and CC in 95% of samples, where in which the prevalence decreased with the degree of the injury [28]. Concerning coinfections, it has been suggested that a synergism exists among the coinfecting HPV genotypes, or contrariwise, different HPV types could compete to colonize the cervical epithelium [10].

Infection with multiple HPV-oncogenic types is prevalent, especially among younger women [30]. The effects of the infection with multiple HPV types on the risk of developing precancerous lesions and cancer are not well understood. In general, it has been assumed that each HPV genotype acts independently; thus, each should contribute individually to the risk of the progress of cytological and precancerous changes [10]. To support this idea, it has been observed that individual histological cervical lesions are usually caused by a single genotype; although, several types are present in the cytology [31]. Under this assumption, we would expect that infection with multiple HPV types produces additive effects to the risk of having abnormal cytology and precancerous cervical lesions. Interestingly, in our samples, we simultaneously detected HR- and LR-HPV, and particularly, LR-HPV were not found alone but were always detected together with HR-HPV genotypes. In our population, we detected six HPV genotypes (HPV-16, HPV-18, HPV-31, HPV-56, HPV-66, and HPV-6) in the biopsy of a patient diagnosed with HSIL. Five of these genotypes are HR-HPVs and one LR-HPV. According to the literature [8, 9, 28], it is likely that, in this case, there is the presence of LR-HPV, entertaining an association with a HR-HPV that may lead to duplication of viral charges and exert a synergistic effect in the oncogenesis.

In this study, we found that coinfection correlated with the injury type, supporting previous reports that afford value to the detection and characterization of multiple HPV infections in clinical samples in order to search for markers for abnormal cervical cells.

Studies of HPV coinfection in Mexico are scarce to date; Aguilar-Lemarroy et al. [32] reported that, in samples diagnosed with CIN-I and CIN-III, the percentage of coinfection was 58.2 and 60%, respectively, whereas the incidence of coinfection in cervical cancer decreased to 26.4%. Similarly, we found a higher percentage of coinfection in LSIL (100%) and ASCUS (88%) than in HSIL and CC (45% and 22%, respectively). Thus, the presence of coinfection increased, to a greater extent, the chance of developing LSIL (OR = 41, *P* = 0.02) than HSIL (OR = 18.6, *P* = 0.05) or cervical cancer (OR = 5.6, *P* = 0.05). This result supports the hypothesis that the subsequent accumulation of different infections can be a marker of persistent infection and, eventually, a possible risk factor for the progression of the lesion.

Another key factor in the development of cervical lesions is the presence of simple or mixed coinfections [3]. We observed a high frequency of mixed coinfections (HR-HPV + LR-HPV) in LSIL and ASCUS (70% and 65%, respectively) and severe injuries such as HSIL and cervical cancer, and a higher frequency of simple coinfections (HR-HPV + HR-HPV) (100% and 80%, respectively).

It is noteworthy that 36% of the observed coinfection contains HPV-16; similarly, López-Rivera et al. [20] reported that this viral genotype is present in 43% of the coinfected population. Despite this, we observed high single infections containing HPV-16 (13/25 HPV-16 positive). Another important datum of our work is that HPV-31 was found only in coinfections.

Regarding results about the adjacent regions without morphological changes, the prevalence of HPV infection (80%) was similar to that found in lesion biopsies. However, coinfection was low (30%) in the adjacent areas without morphological changes of patients with cervical lesions; although, all of these HPV-positive areas could develop some injury over time, considering that the prevalence of HPV-16 in these regions was 100%. Bearing these data and the OR results (HPV-16/CC, OR = 10.3, *P* ≤ 0.05) in mind, we were able to predict that all these regions have a 91% chance of developing cervical cancer. It is noteworthy that these results are not comparable with those found in the literature, because majority of studies only genotypify the HPV found in intraepithelial injuries, but not in the areas adjacent to the cervical lesion. In this regard, it is important to note that these results highlighted the importance of studies about viral genotyping, even if patients do not exhibit any injury by colposcopy, as it would aid in timely diagnosis and appropriate treatment for the prevention of the development of intraepithelial lesions and cervical cancer. In recent years, several epidemiological studies have mentioned the presence of single infections and coinfections in control groups. For instance, Aguilar-Lemarroy [32] reported that 68.8% of healthy controls are infected with HPV-16 and a high percentage of coinfections with other HPV. These data and our results demonstrate that, in addition to HPV-16 (the most prevalent HPV worldwide), it is necessary to pay attention to coinfection in conjunction with genotypes that, although are less frequent, they may entertain local epidemiological importance. The high frequency of HR-HPV in cases of LSIL and HSIL emerges as a reasonable explanation for the high incidence of cervical cancer in our region. Also, determining the frequency and distribution of HPV genotypes can help to measure the effectiveness of an eventual vaccine in our community and aid in streamlining the resources of our national cervical cancer-screening program.

In summary, our data demonstrate the incidence of multiple HPV-type infections in the different grades of the cervical lesion. These data may help to develop better clinical strategies against cervical lesions caused by HPV coinfections and in the search for a more efficient treatment.

## 5. Conclusions

A total of 87.27% of Mexican patients with cervical lesions studied here demonstrated HPV infection, and the most frequent genotypes were HPV-16, HPV-31, HPV-51, and HPV-18. The prevalence of HPV coinfection in the cervical lesions was 70.83%, and coinfection with certain types correlates with the type of cervical lesion, determining that these factors can be used as a predictor of the degree of the injury; in particular, HPV-16 increases the chance to develop cervical cancer. Moreover, HPV detection in healthy areas could be a predictor for the development of injury, given that prevalence of infection in these regions was 80%.

## Figures and Tables

**Table 1:** HPV prevalence.

Samples HPV positive	HR-HPV positive	LR-HPV positive	HPV-18 positive	HPV-16 positive
Single infection	14	0	1	13
Mixed coinfection	19	19	5	5
Simple coinfection	13	2	2	7
All samples HPV-positive (*n* = 48)	46	21	8	25

**Table 2:** Relationship between injury degree and coinfection.

ASCUS	Single infection	Coinfection	OR = 3.5000	Probability
Lesion	1	7	95% CI (0.3884-31.5121) *Z* = 1.117 *P* = 0.2641	^∗∗∗^
Other cervical lesions	13	27		
LSIL	Single infection	Coinfection	OR = 41.0000	Probability
Lesion	0	20	95% CI (2.2597-743.9164) *Z* = 2.511 *P* = 0.0120	97.6%
Other cervical lesions	14	14		
HSIL	Single infection	Coinfection	OR = 0.2299	Probability
Lesion	6	5	95% CI (0.0555-0.9527) *Z* = 2.027 *P* = 0.0427	18.6%
Other cervical lesions	8	29		
CC	Single infection	Coinfection	OR = 0.0625	Probability
Lesion	7	2	95% CI (0.0106-0.3675) *Z* = 3.067 *P* = 0.0022	5.8%
Other cervical lesions	7	32		

**Table 3:** Relationship between of degree injury and HPV type.

CC	Frequency	HPV-16	Other HPV	OR = 10.3529	Probability
Lesion	89.9%	8	1	95% CI (1.1784-90.9562) *Z* = 2.108 *P* = 0.0350	91%
Other cervical lesions		17	22		
LSIL	Frequency	HPV-31	Other HPV	OR = 5.5556	Probability
Lesion	40%	8	12	95% CI (1.2560-24.77) *Z* = 2.248 *P* = 0.0245	85%
Other cervical lesions		3	25		
LSIL	Frequency	HPV-51	Other HPV	OR = 7.0000	Probability
Lesion	35%	7	13	95% CI (1.2702-38.5756) *Z* = 2.235 *P* = 0.254	88%
Other cervical lesions		2	26		

Data obtained with the MedCalc® statistical program.

**Table 4:** HR-HPV prevalence and coinfection in HPV-positive samples without morphological changes areas adjacent to cervical lesions.

Areas without morphological changes adjacent to:	HR-HPV positive (*n* = 20)	Single infection	Coinfection	HPV-16 positive (*n* = 20)	Two HR-HPV genotypes (*n* = 6)	More than two HR-HPV genotypes (*n* = 6)
LSIL (*n* = 2)	2	0	2	2	2	0
HSIL (*n* = 10)	10	6	4	10	3	1
CC (*n* = 8)	8	8	0	8	0	0
Total *n* (%)	20 (100%)	14 (70%)	6 (30%)	20 (100%)	3 (83.33%)	1 (16.66%)

## Data Availability

All data generated of analysis during this study are included in this published article.
